# Electronic Health Record-based COVID-19 Interprofessional Case Collaboration

**DOI:** 10.5811/westjem.22.2.53886

**Published:** 2022-09-14

**Authors:** Bryce Ringwald, Suhair Shawar, Camilla Curren, Jennifer McCallister, Jose Bazan, Christopher Mead, Nicholas E. Kman

**Affiliations:** * The Ohio State University College of Medicine, Columbus, Ohio; † The Ohio State University College of Medicine, Department of Internal Medicine, Columbus, Ohio; ‡ The Ohio State University College of Medicine, Department of Pharmacy, Columbus, Ohio; § The Ohio State University College of Medicine, Department of Rehabilitation Sciences, Columbus, Ohio; ¶ The Ohio State University College of Medicine, Department of Emergency Medicine, Columbus, Ohio

## BACKGROUND

The Coronavirus Disease 2019 (COVID-19) pandemic led many health science colleges to remove their students from the clinical environment or discourage their involvement with COVID-19 to ensure safety.^1^ Additionally, the therapeutics literature for COVID-19 is rapidly evolving.^2^ As colleges determine student involvement in the care of patients with COVID-19, it is imperative to teach evidence-based approaches to management in an interprofessional manner before students begin caring for this patient population.

We developed an interprofessional case collaboration between medical and pharmacy students demonstrating a management plan based on the most current evidence-based approaches. Students collaborated on two clinical cases of COVID-19 pneumonia in an electronic health record (EHR) training environment.^3^ Students also discussed medical misinformation, scarce resource allocation, and interprofessional collaboration.

Herein, we describe how one institution piloted this curriculum into the fourth-year Emergency Medicine (EM) clerkship to prepare students for the evaluation of patients with suspected or confirmed COVID-19. We report the development, feasibility, experiences, and strategies for implementation at other academic institutions.

## OBJECTIVES

By the end of this case collaboration, students should be able to:

1. Describe the therapeutic management of COVID-19 pneumonia
2. Collaborate with interprofessional team members to create a care plan
3. Describe an approach to address medical misinformation
4. Appreciate the ethical allocation of scarce resources

## CURRICULAR DESIGN

### Curricular needs assessment

Like many health science colleges, students at our institution were removed from clinical duties in the first week of March 2020 to ensure learner safety. Upon reintroduction to the clinical environment in June 2020, students were initially restricted from the care of patients with suspected or confirmed COVID-19. Without clinical experience, students will not have the opportunity to participate in the direct collaborative care of patients with COVID-19 before entering residency.

### Curriculum development

Kern’s six-step approach^4^ (Table 1) was utilized to develop an EHR-based, interprofessional, COVID-19 management curriculum. The curriculum was implemented on three separate occasions with cases being revised based on frequently changing organizational guidelines and learner feedback. Two cases of patients with COVID-19 pneumonia were created by EM, pulmonary/critical care, infectious disease, pharmacy, and respiratory therapy providers. One case (“Jane Covid”) simulated a patient with moderate COVID-19 pneumonia with few comorbidities, but stable for discharge from the emergency department (ED) and thus a candidate for monoclonal antibody therapy based on current guidelines. The second case (“Joe Covid”) simulated a patient with severe COVID-19 pneumonia with multiple comorbidities requiring critical care, and thus a candidate to receive oral dexamethasone with or without intravenous remdesivir based on the collaboration. Cases were built into an EHR training environment.^4^

### Pre-collaboration reading and case review

Fourth-year medical and pharmacy students were assigned to read our institutional clinical guidelines and current literature on dexamethasone^5^, remdesivir^6^–^8^, bamlanivimab^9^, and casirivimab/imdevimab^10^, and regarding combating medical misinformation.^11^ Learners were encouraged to review additional literature such as evidence against the use of remdesivir.^7^,^8^ Medical students were assigned one of the two cases to review prior to the collaboration.

### Case collaboration

Medical students, pharmacy students, and faculty were separated into small groups with at least one representative from each discipline in Zoom^12^ breakout rooms. Medical students presented one of the patient cases to the group and consulted pharmacy students, serving as therapeutic consultants, for guidelines based on institutional standards of care. Groups discussed the patient’s disposition and management options. These same steps were repeated for students presenting the second patient case. Finally, group discussions regarding medical misinformation, interprofessional collaboration, and scarce resource allocation were led by the faculty members. Students were assessed based on participation, professionalism, and collaborative skills by their interprofessional peers and faculty.

### Post-collaboration assessment

Students completed a worksheet with questions regarding management of patients with COVID-19. Worksheets were assessed for accuracy by a rubric.

## IMPACT/EFFECTIVENESS

This interprofessional case collaboration on the management of patients with COVID-19 pneumonia was piloted three times with a total of 21 fourth-year medical students on their EM clerkship or as part of a transition to EM residency course and 5 pharmacy students on their EM clinical rotation, divided among five small group case collaborations. 15 medical students and 5 pharmacy students evaluated the exercise. Both medical students and pharmacy students rated the overall quality of the session as Very Good (4.0/5.0). They rated the instruction as Excellent (4.6/5.0).

## DISCUSSION

We successfully utilized the EHR training environment^4^ to create an interprofessional case collaboration in the ED digital setting. This format allowed students to interact with the EHR by reviewing the patient chart, placing orders, simulating working conditions, and utilizing organization-specific checklists for medication appropriateness. In addition, we allowed students to examine conflicting evidence^6^–^8^ regarding the utilization of certain medications requiring the students to collaborate and appraise the evidence before deciding on care plans. Discussions at the end of the collaboration on medical misinformation, interprofessional collaboration, and scarce resource allocation offered rich insight into front-line experience.

We continue to improve the quality of this COVID-19 clinical case curriculum. We plan to include the utilization of screen sharing on webinar-based platforms to review the chart and guide students through their case presentation *in situ*. Additionally, we plan and invite faculty and pharmacy students from other rotations such as infectious disease and critical care to participate. We continue to necessarily revise the cases to stay up to date with the changing evidence surrounding management of COVID-19. Finally, it was evident that the collaboration was not as robust when medical or pharmacy students had not completed the pre-work, so this will be emphasized.

For this program, we built the patient cases and chart elements into a simulated EHR training environment^4^; however, these cases can be utilized with a paper chart format to achieve the same result. We utilized our institution’s treatment guidelines as a template for therapeutic management decision-making. Health science colleges with multiple hospital affiliations could instead utilize Infectious Disease Society of America^13^ or National Institute of Health^14^ treatment guidelines as a generic guide to develop their therapeutic criteria.

## Figures and Tables

**Table 1:** Utilization of Kern’s six-step approach for curricular development.

Kern’s six-step approach	Utilization in curriculum development
Step 1: Problem identification and general needs assessment	Fourth-year medical students have limited contact in care for patients with COVID-19 for concerns of safety
Step 2: Targeted needs assessment	AAMC recommendations for medical students to participate in virtual care of patients with COVID-19^1^
Step 3: Goals and objectives	Describe the therapeutic management of COVID-19 pneumonia Collaborate with interprofessional team members to create a care plan Describe an approach to address medical misinformation Appreciate the ethical allocation of scarce resources
Step 4: Educational strategies	- Reading for medical and pharmacy students^5^–^11^ - Pre-collaboration case EMR interaction - Interprofessional case collaboration - Post-collaboration worksheet
Step 5: Implementation	- Zoom platform breakout sessions^12^ - Small group sessions of medical students, pharmacy students, and faculty
Step 6: Evaluation and feedback	- Interprofessional peer evaluation and faculty evaluation of collaboration - Evaluation of worksheets for accuracy - Overall curriculum evaluation

*AAMC*, Association of American Medical Colleges; *EMR*, electronic medical record.
